# Causal relationships between CD25 on immune cells and hip osteoarthritis

**DOI:** 10.3389/fimmu.2023.1247710

**Published:** 2023-09-04

**Authors:** Hao Luo, Yong Zhu, Bin Guo, Zhe Ruan, Zhi Liu, Zhihua Fan, Shushan Zhao

**Affiliations:** ^1^ Department of Orthopaedics, Xiangya Hospital, Central South University, Changsha, China; ^2^ National Clinical Research Center for Geriatric Disorders, Xiangya Hospital, Central South University, Changsha, Hunan, China; ^3^ Department of Dermatology, Xiangya Hospital, Central South University, Changsha, China

**Keywords:** hip OA, CD25, SNP, Mendelian randomization, causal relationship

## Abstract

**Objectives:**

Previous research has indicated a potential association between immune factors and osteoarthritis (OA), but the causal relationship between CD25 expression on immune cells and hip OA remains enigmatic. To shed light on this relationship, this study utilized the two-sample Mendelian Randomization (MR) method.

**Methods:**

Leveraging genome-wide association studies (GWAS) data from the UK Biobank and arcOGEN, the investigation encompasses a substantial European cohort comprising 15,704 hip OA cases and 378,169 controls. Genetic insights into CD25 stem from a subgroup of 3,757 individuals with European ancestry, encompassing 77 CD25-related traits. Several MR methods were applied, and robustness was assessed through heterogeneity and sensitivity analysis.

**Results:**

Among the 77 traits examined, 66 shared the same single nucleotide polymorphisms (SNPs) with hip OA. Of these, 7 CD25-related traits were found to be causally associated with hip OA (adjusted *P*><0.05), with F-statistics ranging from 33 to 122. These traits are specifically related to CD4^+^CD25^+^ T cells, exhibiting odds ratios (OR) and 95% confidence intervals (CI) less than 1. Notably, no causal link was discerned with the CD8+CD25+ T cell subset. Within absolute count (AC) and relative count (RC) trait types, a significant causal relationship was observed solely between CD4^+^CD25^+^ T cells and hip OA, without subtype localization. A more intricate examination of CD25 expression levels within the CD4^+^CD25^+^ T cell subset revealed a correlation with the CD39+ regulatory T (Treg) subset and hip OA, particularly within the CD39^+^ activated Treg subset. Furthermore, a notable causal relationship emerged between CD25 expression levels in the CD45RA^-^ not Treg subset and hip OA. However, no significant causal link was established with any subsets of B cells.

**Conclusion:**

The genetic prediction suggests that CD25, particularly within the realm of CD4^+^CD25^+^ T cells, may exert a protective influence against the development of hip OA. These findings provide a novel therapeutic approach for the prevention and treatment of hip OA.

## 1 Introduction

Hip osteoarthritis (OA) is an intricate degenerative condition affecting the hip joint, primarily triggered by a combination of chronic inflammatory and metabolic factors. It is characterized by narrowing of the joint space, degeneration of articular cartilage, osteosclerosis of subchondral bone, osteophyte formation, and chronic inflammation (1, 2). The Global Burden of Disease Study 2019 underscores a gradual rise in the incidence of hip OA, attributed to the aging population, consequently heightening the risk of disability and inflicting substantial societal and patient-related burdens (3–5). Nevertheless, studies on the etiology and therapeutic avenues for hip OA have not kept pace with those for knee OA. This discrepancy may be attributed to the higher prevalence of knee OA, resulting in the direct extrapolation of etiological and clinical insights from knee OA studies (6). However, this may lead to the neglect of other causes that contribute independently to hip OA. While a comprehensive understanding of hip OA’s pathogenesis remains elusive, certain studies have validated the intricate connection between immune-related factors and the emergence of both knee and hip OA (7–10). In light of this context, we propose a hypothesis asserting the pivotal role of immune-related elements in the pathophysiology of hip OA, thereby offering a novel framework to explore its underlying etiological factors and potential avenues for therapeutic intervention.

CD25, the α chain of interleukin-2 receptor (IL-2R), is predominantly expressed on the surface of immune-related cells, including activated T cells, B cells, and NK cells (11) CD25 forms a high-affinity heterotrimeric receptor alongside β and γ chains, engaging in diverse immune responses upon interaction with the ligand IL-2 (12–14). Furthermore, CD25 on immune cells has been implicated in various immune and inflammatory diseases across multiple studies. Research has shown that low expression of CD4^+^CD25^+^ regulatory T cells (Tregs) in patients with rheumatoid arthritis suggests that immune suppression mediated by CD25^+^ Tregs is an important mechanism in the development of rheumatoid arthritis (15, 16). In a separate study exploring factors contributing to periodontitis, CD25 was found to promote the immune inhibitory effects of Tregs and B cells, thereby safeguarding periodontal tissues from inflammation-induced damage (11). The influence of CD25 within the tumor microenvironment has also been extensively investigated, emphasizing the potential of mitigating Treg-mediated immune suppression as a pivotal facet of tumor immunotherapy, positioning CD25 as a viable target for immunotherapeutic strategies (17–19). Moreover, CD25 plays an important role in mediating transplant tolerance and other autoimmune diseases such as atopic dermatitis, Hodgkin’s lymphoma, etc (14, 20). Although there is existing evidence suggesting the role of immune cells in the development of hip OA (21, 22), the precise impact of CD25 on immune cells in this context remains uncharted territory. The causal link between CD25 on immune cells and hip OA still lacks clarity, underscoring the need for comprehensive further research.

Within the realm of epidemiological research, conventional observational studies frequently introduce bias to research outcomes as a result of confounding variables and the potential for reverse causation, thereby diminishing their overall credibility. Conversely, the execution of randomized controlled trials (RCTs) encounters an array of practical limitations that render their implementation within clinical environments a formidable undertaking (23). Mendelian randomization (MR) emerges as an analytical methodology aimed at examining potential causal links between exposures and outcomes, utilizing data derived from genome-wide association studies (GWAS) (23–28). MR uses exposure-related genetic variations as instrumental variables (IVs) and follows the Mendelian gamete random allocation principle as well as the principle of free combination. This approach effectively mitigates the impact of confounding factors and reverse causation commonly encountered in conventional observational studies (29, 30). Thus, based on the available GWAS data on CD25 on immune cells and hip OA, the genetic variants of CD25 on immune cells were investigated as IVs in this study using a two-sample MR approach to investigate whether there is a causal association between CD25 on immune cells and hip OA.

## 2 Material and method

In this research, a two-sample MR method was employed to conduct a causal association analysis. CD25 on immune cells served as the exposure variable, and single nucleotide polymorphisms (SNPs) that showed a significant association with CD25 on immune cells were employed as IVs. And the outcome of interest centered around hip OA. Heterogeneity Analysis and sensitivity analysis were conducted to ensure the robustness of the results.

### 2.1 Data sources

A secondary analysis was conducted on existing data to identify dependable exposure and genetic variations available in publicly accessible databases. Consequently, no additional ethical approval was required. Considering that the frequency of fingerprint genetic variants varies among populations with different genetic backgrounds, leading to spurious associations between genetic variants and outcomes, we opted to focus our analytical framework on European populations within both samples.

To investigate the potential impact of CD25 on immune cells on hip OA, GWAS data for CD25 on immune cells and hip OA were obtained at the ieu open GWAS project website (https://gwas.mrcieu.ac.uk/datasets). The genetic variation for CD25 on immune cells was derived from a cohort of 3,757 Europeans (31). From this cohort, a total of 77 CD25-related traits were obtained, and all the data included in the analysis were obtained through Flow Cytometry. These 77 traits were categorized into three groups: Absolute count (AC), Relative count (RC), and Median Fluorescence Intensity (MFI). Among them, there were 14 traits in the AC group, representing the total number of cells in the analyzed subgroup. The RC group consisted of 28 traits, representing the proportion of cells in the analyzed subgroup compared to other cell groups. For example, CD25^hi^ CD4^+^ T cell %CD4^+^ T cell is a relative value. The MFI group included 35 traits, which indirectly reflected the expression levels of CD25 protein in various subgroups of cells. The fluorescence intensity of CD25 expression can increase either by having more cells expressing CD25 or by having a higher expression level of CD25 in individual or specific subgroups of cells, even when the total cell count remains constant. **Supplementary Table S1** presents the GWAS IDs of CD25-related traits, the name of the traits represented, the trait type, etc.

Moreover, genetic data on hip OA were obtained from the UK Biobank and Arthritis Research UK Osteoarthritis Genetics (arcOGEN) resources for a large European population, involving 15,704 cases and 378,169 controls, which included approximately 22 million SNPs, and the GWAS ID of hip OA is GCST007091 (32).

### 2.2 Selection of IVs

3 core assumptions must be met for SNPs to serve as IVs. First of all, correlation: the prerequisite of correlation necessitates that SNPs demonstrate a robust correlation with the exposure of interest. Notably, the strength of IVs, as measured by F values, should be set at a minimum threshold of ≥10. A value below 10 may potentially introduce significant bias into estimates of causal effects (33). Afterward, exclusivity: the principle of exclusivity mandates that IVs remain devoid of any direct association with the outcome. Their influence should be solely mediated through exposure, ensuring the absence of genetic pleiotropy. In the end, independence: the principle of independence dictates that IVs remain unlinked to confounding factors that exert an impact on both the exposure and the outcome (34). MR capitalizes on the stochastic segregation and recombination of genetic variants during gamete formation, thereby emulating a process of random assignment within a population. Importantly, this procedure typically remains uncorrelated with external environmental confounders, bolstering the integrity of MR analyses (35–37).

To ensure the authenticity and reliability regarding the causal relationship between CD25 on immune cells and hip OA, the following parameters were employed to screen the optimal IVs. Firstly, noteworthy SNPs were meticulously screened from the GWAS database of CD25-related traits. and the F statistic was set to F≥10, corresponding to the significance level of the “IV-exposure” association, commonly acknowledged as a robust screening threshold for IVs. In addition, a requirement for sufficient IV strength was upheld, with a screening threshold of *P*<5×10-8, effectively preempting potential biases originating from weak IVs. Secondly, the linkage disequilibrium (LD) coefficient was set as r^2^ of 0.001 and the maximum LD region span of 10,000kb was set. This meticulous parameterization ensured the independence of the selected SNPs and effectively mitigated any LD-related complications. Thirdly, leveraging information from the exposed and extracted SNPs, an explicit protocol was enacted. A minor allele frequency (MAF) threshold of 0.05 was established, and proxy SNPs were strategically employed for SNPs with missing data. SNPs devoid of alternative loci were systematically eliminated. Subsequently, the SNPs identified based on CD25-related traits were extracted from the GWAS database for hip OA. Finally, information from the above two datasets was pooled and excluded SNPs with palindromic structures and MAF > 0.42.

### 2.3 Statistical analyses

All data were statistically analyzed using R software version R-4.2.2. And all estimates were bilaterally ≤0.05 significant level. In this study, an array of methodologies was employed to infer the potential causal effects of CD25-related traits on hip OA, encompassing the Wald ratio, inverse-variance weighted (IVW), MR-Egger, simple mode, and weighted mode methods. If only one SNP was identified for a significant trait in both databases, only the Wald ratio approach was utilized for analysis. When there were two or more SNPs, the IVW approach was applied. For cases where there were three or more SNPs, the IVW and MR-Egger approaches were implemented. Assessment of SNP heterogeneity across the two samples was conducted via Cochran’s Q test, with significance established at *P* < 0.05 to indicate noteworthy heterogeneity. In cases where heterogeneity and pleiotropy were absent, precedence was given to the results derived from the IVW approach. In the presence of heterogeneity, the random effects model of IVW could be invoked. However, if pleiotropy was detected, preference was granted to the outcomes derived from MR-Egger calculations (38). To prevent type I errors that arise from multiple hypothesis testing, the Benjamini-Hochberg method of False Discovery Rate (FDR) was employed to adjust the *P* value and control the overall error rate. In the MR analysis, the beta value is commonly used to estimate the causal effect of genetic variation on the exposure and outcome. We convert the beta value to the odds ratio (OR) using the formula OR=exp(beta). The OR is a measure of association between the exposure and outcome, providing a more intuitive understanding of the actual implications of the results. If OR=1, it indicates that the exposure does not affect the odds of the outcome. If OR>1, it suggests that the exposure is associated with higher odds of the outcome. Conversely, if OR<1, it indicates that the exposure is associated with lower odds of the outcome.

The MR-Egger regression analysis was utilized to assess potential horizontal pleiotropy effects attributed to the encompassed SNPs. Should the intercept term of the MR-Egger method significantly deviate from 0, this would signal the presence of horizontal pleiotropy. Additionally, sensitivity analysis was performed using the Leave-one-out sensitivity test, whereby the outcomes were recalculated following the stepwise removal of individual SNPs. The objective was to ascertain whether the exclusion of any SNP yielded a substantial alteration in the results. The robustness of the MR findings was established if no SNP omission yielded a significant impact on the overall outcomes.

In addition, the chosen SNPs underwent a comprehensive cross-validation process using the website http://www.phenoscanner.medschl.cam, aimed at identifying any additional traits that could exert an influence on the study outcomes. SNPs found to exhibit associations with these additional traits were subsequently excluded, effectively minimizing the potential influence of confounding factors.

## 3 Results

### 3.1 Selection of IVs

After a series of strict quality control, it was determined that among the 77 CD25-related traits, a total of 66 exhibited an intersection with SNPs sourced from the large-scale hip OA GWAS. However, only 7 of these CD25-related traits had a significantly adjusted *P*-value <0.05. Notably, the F-statistics for these 7 CD25-related traits ranged from 33 to 122, ensuring the strength of the IVs and avoiding bias due to weak instruments. The preliminary screened SNPs were obtained after removing LD effects, but one SNP (rs6584030) with palindromic structure and MAF > 0.42 was excluded. Comprehensive details about the SNPs, including SNP ID, beta value, standard error (se), *P*-value, effect allele (EA), and other allele, were systematically summarized for subsequent analysis, as shown in **Supplementary Table S2**.

### 3.2 Two-sample MR analysis

As shown in **Table 1**, the transformation of beta values into OR via the formula OR=exp(beta) was executed, accompanied by the computation of 95% confidence interval (CI). A notable association emerges, indicating that 7 CD25-related traits are intricately linked with the susceptibility to hip OA, serving as protective elements against its development. All 7 CD25-related traits in the panel were Tregs, and they were classified into three different trait types: AC, RC, and MFI. AC and RC denote the quantity of a specific cellular subset within CD25^+^, whereas MFI represents the intensity of CD25 expression within a particular cellular subset of CD25^+^.

**Table 1:** Two-sample MR estimates of associations between CD25 on immune cells and hip OA.

Exposure	Trait.type	Panel	Nsnp	SNP	Nearby Gene	Mthod	OR	95%CI	*P*-adjusted	Cochran’s Q Statistic	*Heterogeneity P-value*	MR-Egger intercept (*P*-value)
CD4^+^CD25^hi^T cell AC	AC	Treg	1	rs61839660	IL2RA	Wald_ratio	0.87	0.80-0.94	0.013	–	–	–
CD4^+^CD25^h^iT cell %T cell	RC	Treg	1	rs61839660	IL2RA	Wald_ratio	0.88	0.82-0.95	0.013	–	–	–
CD4^+^CD25^hi^ T cell %CD4^+^T cell	RC	Treg	1	rs61839660	IL2RA	Wald_ratio	0.90	0.84-0.96	0.013	–	–	–
CD25 on CD4^+^T cell	MFI	Treg	1	rs61839660	IL2RA	Wald_ratio	0.84	0.76-0.93	0.013	–	–	–
CD25 on CD39^+^ CD4 Treg	MFI	Treg	1	rs7078614	IL2RA	Wald_ratio	0.80	0.71-0.91	0.024	–	–	–
CD25 on CD39^+^ activated CD4 Treg	MFI	Treg	1	rs7078614	IL2RA	Wald_ratio	0.79	0.69-0.91	0.024	–	–	–
CD25 on CD45RA^-^ CD4 not Treg	MFI	Treg	2	rs181516190	TBX20	IVW	0.90	0.85-0.96	0.039	0.024	0.877	–
				rs61839660	IL2RA							

MR, Mendelian randomization; OA, osteoarthritis; SNP, single-nucleotide polymorphism; OR, odds ratio; CI, confidence intervals; AC, Absolute Count; Treg, regulatory T cell; IVW, Inverse variance weighted."-" indicate that the value does not need to be calculated or measured.

**Figure 1:**
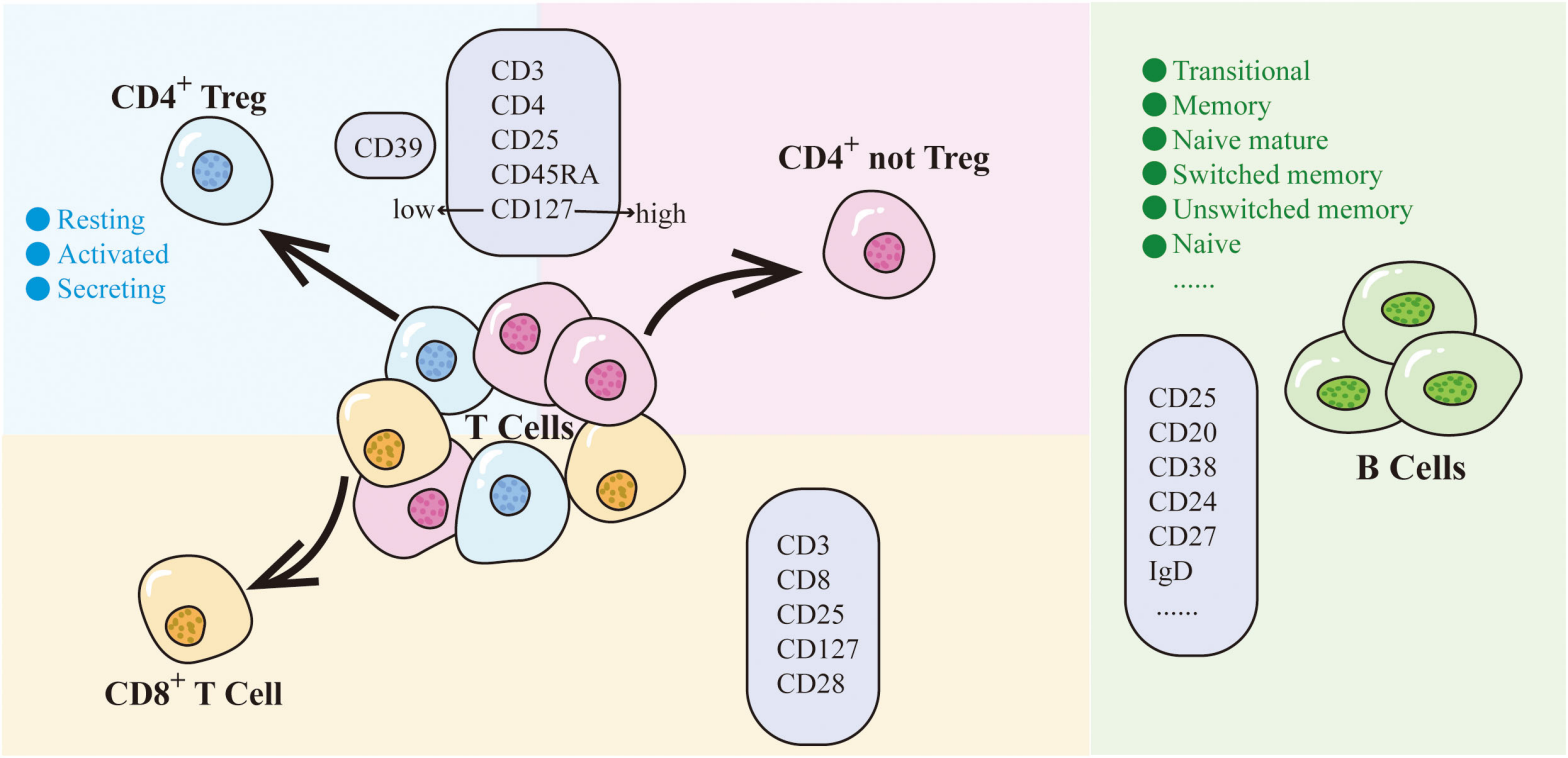
CD25 on T cells and B cells. Different colored backgrounds represent distinct cell subsets. T cells are divided into CD4^+^ subset and CD8^+^ subset. The CD4^+^ subset includes CD4^+^ Treg (light blue) and CD4^+^ not Treg (light pink). CD8^+^ T cells are depicted in light orange, while B cells are represented in green. The light purple box indicates the molecules expressed by cells within that particular subset.

Among the above 7 traits, only CD25 on CD45RA^-^ CD4 not Treg has two SNPs (rs181516190, rs61839660), with analysis conducted via the IVW method. In the heterogeneity evaluation, Cochran’s Q test showed a heterogeneity *P*-value of 0.877, indicating no heterogeneity. The remaining traits, characterized by just one SNP each, underwent analysis using the Wald ratio method, thereby obviating the need for a heterogeneity test. Furthermore, all 7 CD25-related trait SNPs were less than three, so no horizontal pleiotropy testing was needed. Upon comprehensive scrutiny within PhenoScanner, no direct links to the outcome were identified for all SNPs, and they exhibited no affiliation with potential confounding factors influencing the association between exposure and outcome. The subsets and molecular expression of CD25^+^ T cells and B cells in the results are illustrated in **Figure 1**.

#### 3.2.1 Treg: AC,RC trait type

Within the Treg panel, CD25^+^ cells can be classified into 2 main cell subpopulations based on the expression of CD4 and CD8 on the T cell surface: CD4^+^CD25^+^ and CD8^+^CD25^+^. The CD4^+^CD25^+^ traits within the Treg panel were further categorized into two distinct groups, Treg (CD25^hi^CD127^low^) and not Treg (CD25^hi^CD127^hi^), based on CD127 expression levels. Moreover, the CD4^+^CD25^+^ Treg subpopulation was systematically subdivided into 3 subtypes according to their cellular characteristics: activated (CD25^+++^CD45RA^-^), resting (CD25^++^CD45RA^+^), and secreting (CD25^++^CD45RA^-^). An additional layer of classification can be undertaken based on the expression of CD39. Similarly, the CD8^+^CD25^+^ traits can be divided into different subgroups depending on the expression of CD28 and CD127.

The findings demonstrated that CD4^+^CD25^hi^ T cell AC (OR: 0.84, 95% CI: 0.76-0.93, *P*-adjusted=0.013) was significantly associated with hip OA. However, there was no significant causal association found between the AC traits of CD8^+^CD25^+^ cells and hip OA. Furthermore, the ensuing findings failed to indicate AC traits of CD4^+^CD25^+^ Treg (activated/resting/secreting) and CD4^+^CD25^+^ not Treg and hip OA were causally significantly associated with each other. Not only did AC show a significant association with hip OA within the Treg panel, but RC also exhibited a significant association with hip OA. Among the RC-related traits, CD4^+^CD25^hi^ T cell %T cell (OR:0.88,95%CI:0.82-0.95, *P*-adjusted=0.013) and CD4^+^CD25^hi^ T cell %CD4^+^ T cell (OR:0.90,95%CI:0.84-0.96, *P*-adjusted=0.013) indicated a significant causal relationship between the RC traits of CD25 and hip OA. However, no subpopulations within the RC traits of CD4^+^Treg (activated/resting/secreting) or CD4^+^ not Treg (CD45RA^+^/CD45RA^-^) were deemed significantly associated with hip OA. Additionally, no significant causal relationship was identified between any cell subpopulations within the RC traits of CD8^+^CD25^+^ cells and hip OA.

#### 3.2.2 Treg: MFI trait type

In addition to the significant causal relationships observed between the AC and RC traits of CD25 and hip OA, it is pertinent to highlight the link between the expression level of CD25 in immune cells and hip OA. We discovered traits within the CD4^+^CD25^+^ cell subpopulation that exhibit a causal association with hip OA. Specifically, the presence of CD25 on CD4^+^ T cell (OR: 0.84, 95% CI: 0.76-0.93, *P*-adjusted=0.013) was associated with a reduced risk of hip OA. In the realm of Tregs, CD25 on CD39^+^ CD4 Treg (OR: 0.80, 95% CI: 0.71-0.91, *P*-adjusted=0.024) emerged as a significant factor in hip OA. Further scrutiny revealed that within the CD39^+^ subgroup cells, CD25 on CD39^+^ activated CD4 Treg (OR: 0.79, 95% CI: 0.69-0.91, *P*-adjusted=0.024) displayed a close correlation with hip OA. Notably, among the not Treg subset, CD25 on CD45RA^-^ CD4 not Treg (OR: 0.90, 95% CI: 0.85-0.96, *P*-adjusted=0.039) was associated with hip OA. However, no discernible causal relationship between any cell subpopulations within CD8^+^CD25^+^ and hip OA was discerned.

#### 3.2.3 B cells

The presence of CD25 expression on B cells did not exhibit a causal link to hip OA. Within the B cell panel, a delineation into distinct subsets can be achieved based on CD24 versus CD38 expression, thereby encompassing transitional (CD24^+^CD38^hi^), memory (CD24^+^CD38^−/dim^), and naive mature (CD24^−^CD38^−/dim^) subsets. Further stratification based on CD27 versus IgD establishes additional subclasses, including switched memory (CD27^+^IgD^−^), unswitched memory (CD27^+^IgD^+^), naive (CD27^−^IgD^+^), and CD27^−^IgD^−^ B cells. Additionally, classification using IgD versus CD38 lends itself to the identification of Bm1–Bm5 subgroups, and various other B cell subgroups can be discerned based on parameters like CD24 versus CD27, IgD versus CD24, and CD20 versus CD38. However, our findings demonstrated that CD25 expression on B cells, including all CD25^+^ B cell subgroups, was not causally related to hip OA.

## 4 Discussion

Although OA has historically been considered a non-inflammatory degenerative disease, emerging evidence underscores the role of immune-related factors in its pathogenesis, with their active involvement in the inflammatory processes underlying OA. Therefore, the attenuation of anti-inflammatory effects and the enhancement of pro-inflammatory effects can both exacerbate the progression of inflammation, thereby driving the advancement of OA (7–10, 39, 40). CD25, a pivotal immune-related component, serves as the α chain of the IL-2R and is predominantly present on the surface of activated T cells, B cells, and NK cells. Functioning as a critical constituent of the IL-2R, CD25 competes for IL-2 binding, thereby reducing immune responses mediated by free IL-2. Up until now, no studies have used large-scale data to perform an in-depth analysis of the causal relationship between CD25 and hip OA. However, this study employs a two-sample MR analysis based on extensive GWAS data to establish a causal association between CD25 and hip OA, providing evidence for CD25 as a protective factor against hip OA. These findings are consistent with previous research on the role of CD25 in OA. For instance, an exploration into mesenchymal stem cell (MSC) therapy for OA revealed that treatment engendered the accumulation of CD4^+^CD25^+^ leukocyte subsets within the joints, effectively mitigating OA progression (41). Similarly, a distinct study demonstrated that OA patients exhibit lower proportions of CD4^+^CD25^+^ Tregs compared to healthy individuals, potentially contributing to increased pain and functional impairment (39). While previous studies have reported a significant increase in CD8^+^CD25^+^ T cells in patients with rheumatoid arthritis, which exacerbates the progression of inflammation and may be an important pathogenic mechanism (42). However, our findings do not provide evidence of a causal relationship between the CD8^+^CD25^+^ T cell subset and hip OA, suggesting the need for additional research to explore this association.

Based on the results of this MR study, it is evident that all 7 CD25-related traits exhibiting significant correlations with hip OA are specifically related to CD4^+^CD25^+^ T cells. Notably, among the AC and RC trait types, a significant causal relationship between CD4^+^CD25^+^ T cell subgroups and hip OA has been discerned, though the precise subgroup of cells responsible remains to be conclusively identified. Further analysis of CD25 expression levels revealed that both Treg and not Treg subgroups of CD4^+^CD25^+^ T cells play crucial roles in this association. The primary function of CD4^+^CD25^+^ Tregs is to maintain the body’s immune tolerance and suppress the immune response thus maintaining and regulating the body’s immune homeostasis, which is increasingly studied in inflammation, autoimmune diseases, and tumors (14, 16, 17). Consequently, an escalation in the count of CD25-expressing Tregs or elevated CD25 expression levels can enhance Tregs function, which is essential for suppressing the progression of immune inflammation. Remarkably, both the CD4^+^CD25^+^CD39^+^ Treg subset and its CD39^+^ activated Treg subgroup exhibit significant causal relationships with hip OA. CD39 is a cell membrane protein with enzymatic activities that hydrolyzes adenosine triphosphate (ATP) and adenosine diphosphate (ADP) into adenosine monophosphate (AMP), leading to energy depletion and the inhibition of immune reactions in various cells (43, 44). This enzymatic prowess of CD39 is likely instrumental in tempering the inflammatory cascade inherent in hip OA. The CD39^+^ activated Treg subgroup of CD39^+^ Tregs, not only exhibits high CD39 expression but also demonstrates the highest level of CD25 expression among all subgroups. This subgroup effectively suppresses immune reactions of hip OA, highlighting its immunosuppressive role in the disease. In contrast, within the not Treg subset, the presence of CD25 on CD45RA^-^ CD4 T cell subset is significantly correlated with hip OA. Our conjecture posits that this subset, characterized by the absence of CD45RA expression, possibly encompasses non-naïve T cells that might be affiliated with effector T cells or memory T cells. However, due to the heightened CD25 expression, these cells engage in competitive binding with IL-2 and rapidly deploy immunosuppressive effects upon stimulation. Yet, the specific intricacies elucidating the immunosuppressive role of CD4^+^ not Tregs warrant comprehensive further exploration and analysis.

In short, all these traits are associated with the expression of CD25 on CD4^+^ T cells. An increased number of T cells expressing CD25 or an elevated expression level of CD25 both attenuate the inflammatory progression of hip OA. We hypothesize that this effect could be attributed to the heightened presence of CD25, which competitively intercepts IL-2, a pivotal factor essential for the survival of adjacent activated T cells. This interception, in turn, results in the suppression of their proliferation or the initiation of apoptosis. Additionally, it promotes the secretion of soluble negative immune molecules such as IL-10 and TGF-β by Tregs, enabling them to exert immunosuppressive effects. Consequently, these mechanisms collectively serve to dampen the inflammatory reaction and impede the progression of hip OA.

The 7 traits encompassing SNPs correspond to 2 genes, rs7078614, and rs61839660 are the IVs of *IL2RA* and rs181516190 is the IV of *TBX20*. *IL2RA* encodes the IL2R-α chain (CD25), which is crucial for the formation of the IL-2/IL-2R signaling pathway participation in several immune activities. It is an important functional gene related to immune-related factors (12, 45). Therefore, the polymorphisms in *IL2RA* are closely associated with hip OA.

*TBX20*, on the other hand, is also an encoding gene that produces a transcription factor belonging to the T-box family. The protein encoded by *TBX20* is involved in regulating developmental processes, particularly in cardiac development, as a conserved master regulator of cardiac development and function (46, 47). While there are no specific reports on the involvement of *TBX20* in hip OA, its polymorphisms suggest a potential link with the pathogenesis of hip OA and its potential as a therapeutic target. Yet, a comprehensive comprehension of the precise mechanisms necessitates further in-depth investigation.

This two-sample MR analysis aims to analyze the causal relationship between CD25 on immune cells and hip OA using available large-sample GWAS data, avoiding the effects of confounding factors and reverse causation and reducing the limitations of traditional observational studies. Furthermore, MR can also alleviate the representativeness and feasibility issues associated with RCTs. Nevertheless, there are some limitations in this study. Firstly, this study relies on publicly available GWAS data, which hinders the possibility of subgroup analysis considering factors like gender, age, and BMI to further explore the effects of other relevant factors on hip OA (26). Secondly, given that the dataset exclusively represents a European population, generalizing the findings to other ethnic groups should be done cautiously, necessitating further scrutiny (27). Thirdly, this study had a limited number of SNPs, and some of the CD25-related traits were associated with only one SNP. This could lead to an overestimation of the relationship between genetic variation and exposure, potentially influenced by the winner’s curse and leading to biased results (48). Finally, all CD25-related traits examined in this analysis were linked to the Treg panel, whereas prior research has indicated a significant role for CD25^+^ B cells in the inflammatory response as well (11, 49). Therefore, further research is necessary to investigate whether CD25^+^ B cells and CD25^+^ NK cells are related to hip OA. Fortunately, the F-statistic value of the IVs in this study was greater than 10, which prevents the potential bias resulting from weak IVs.

In conclusion, this two-sample MR study revealed a causal relationship between CD25 on immune cells and hip OA, particularly CD4^+^CD25^+^ T cells, providing genetic evidence for CD25 as a protective factor for hip OA. It provides a new therapeutic strategy for the treatment of hip OA, making CD25 a potential target for the prevention and treatment of hip OA.

## Data availability statement

The original contributions presented in the study are included in the article/**Supplementary Material**. Further inquiries can be directed to the corresponding author.

## Ethics statement

Ethical approval was not required for the study involving humans in accordance with the local legislation and institutional requirements. Written informed consent to participate in this study was not required from the participants or the participants’ legal guardians/next of kin in accordance with the national legislation and the institutional requirements.

## Author contributions

SZ, HL, and YZ conceptualized and designed the study. SZ, HL, YZ, BG, ZR, ZL, and ZF performed material preparation, data collection, and analysis. SZ, HL, and YZ analyzed and interpreted the data, prepared the original draft, and reviewed and edited the final draft. All authors contributed to the article and approved the submitted version.
